# The application of resveratrol to mesenchymal stromal cell-based regenerative medicine

**DOI:** 10.1186/s13287-019-1412-9

**Published:** 2019-10-17

**Authors:** Chenxia Hu, Lanjuan Li

**Affiliations:** Collaborative Innovation Center for the Diagnosis and Treatment of Infectious Diseases, State Key Laboratory for the Diagnosis and Treatment of Infectious Diseases, First Affiliated Hospital, School of Medicine, Zhejiang University, Hangzhou, 310000 People’s Republic of China

## Abstract

Currently, the transplantation of mesenchymal stromal cells (MSCs) has emerged as an effective strategy to protect against tissue and organ injury. MSC transplantation also serves as a promising therapy for regenerative medicine, while poor engraftment and limited survival rates are major obstacles for its clinical application. Although multiple studies have focused on investigating chemicals to improve MSC stemness and differentiation in vitro and in vivo, there is still a shortage of effective and safe agents for MSC-based regenerative medicine. Resveratrol (RSV), a nonflavonoid polyphenol phytoalexin with a stilbene structure, was first identified in the root extract of white hellebore and is also found in the roots of *Polygonum cuspidatum*, and it is widely used in traditional Chinese medicine. RSV is a natural agent that possesses great therapeutic potential for protecting against acute or chronic injury in multiple tissues as a result of its antioxidative, anti-inflammatory, and anti-cancer properties. According to its demonstrated properties, RSV may improve the therapeutic effects of MSCs via enhancing their survival, self-renewal, lineage commitment, and anti-aging effects. In this review, we concluded that RSV significantly improved the preventive and therapeutic effects of MSCs against multiple diseases. We also described the underlying mechanisms of the effects of RSV on the survival, self-renewal, and lineage commitment of MSCs in vitro and in vivo. Upon further clarification of the potential mechanisms of the effects of RSV on MSC-based therapy, MSCs may be able to be more widely used in regenerative medicine to promote recovery from tissue injury.

## Introduction

The transplantation of mesenchymal stromal cells (MSCs) has emerged as an effective strategy to protect against tissue and organ injury. According to the ISCT criteria, MSCs must have multipotency to differentiate into somatic cells, including osteocytes, adipose cells, and chondrocytes [1]. MSCs with multilineage potential exist prevalently in almost all tissues, and they are promising cell sources for treating multiple diseases without ethical issues [2]. After transplantation in vivo, MSCs exert pro-angiogenic, anti-apoptotic, and antioxidative effects on host tissues and activate local quiescent stem cells to establish cellular interactions by autocrine and paracrine pathways [3]. MSCs are capable of modulating the innate immune system and influencing the functions of T and B cells, including regulatory cells, along with influencing Th1 and Th2 cell differentiation [4–6]. Other advantages of MSCs are their immune privilege and relative safety when used in allogeneic hosts [4]. In addition, homotypic and heterotypic cell fusions of MSCs, although rare, also contribute to the repair of tissues [7]. MSC transplantation also serves as a promising therapy for regenerative medicine, but poor engraftment and limited survival rates are major obstacles to its clinical application. Although multiple studies have focused on investigating chemicals to improve MSC stemness and differentiation in vitro and in vivo, there is still a shortage of effective and safe agents for MSC-based regenerative medicine.

Resveratrol (RSV), a nonflavonoid polyphenol phytoalexin with a stilbene structure, was first found in the root extract of white hellebore (*Veratum grandiflorum*) and was also found in the roots of *Polygonum cupsidatum* in 1963, and it is widely used in traditional Chinese medicine [8]. A large amount of RSV can be found in multiple plants, including peanuts, eucalyptus, blueberries, cranberries, and grapes [9]. *Cis*-RSV is an unstable and less common compound, while *trans*-RSV is the stable form and is more predominant; *trans*-RSV can be converted to *cis*-RSV after exposure to sunlight or UV radiation [10]. RSV effectively inhibits the generation of reactive oxygen species (ROS)/reactive nitrogen species (RNS) and scavenges excessive ROS/RNS mainly through synergistic effects between the 4-hydroxyl group, 3-hydroxyl group, and 5-hydroxyl group [11]. In addition, RSV significantly enhances the interaction between lamin A and SIRT1 [12], which is a member of a family of NAD^1^-dependent deacetylases that participate in the modulation of apoptosis, DNA repair, oxidative stress resistance, anti-aging processes, and lipid metabolism [13]. Several derivatives, such as methoxylated, hydroxylated, and halogenated forms of RSV, have been developed for diverse therapeutic applications [14]. Moreover, RSV is a natural agent that possesses a wide therapeutic potential as a result of its antioxidative [15], anti-inflammatory [16], and anti-cancer [17] properties for the protection of multiple tissues against acute or chronic injury. According to its demonstrated properties, RSV may improve the therapeutic effects of MSCs by enhancing their survival, self-renewal, lineage commitment, and anti-aging effects.

**Fig. 1:**
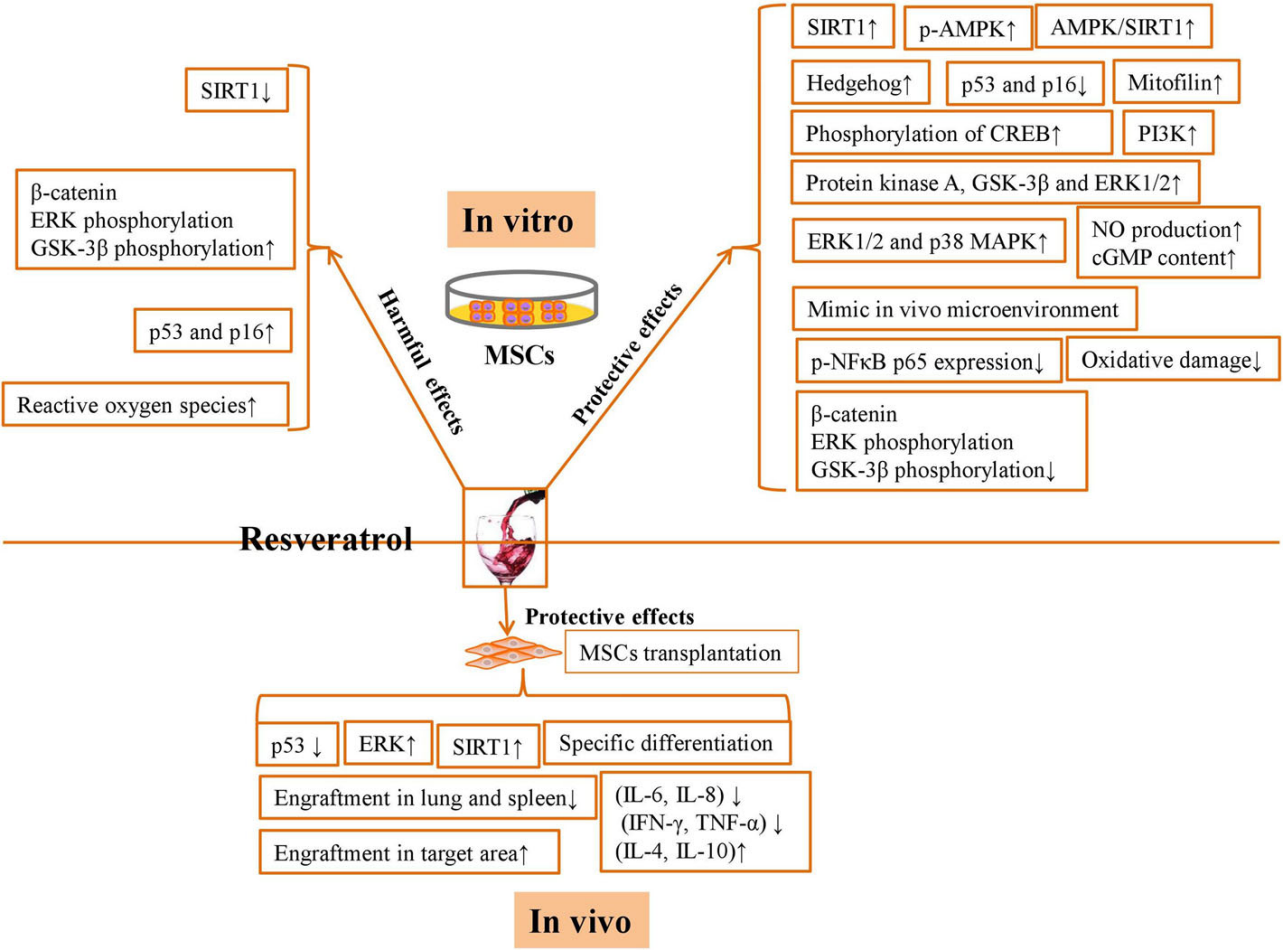
The underlying mechanisms of the protective and harmful effects of RSV on MSCs in vitro and in vivo

In this review, we concluded that RSV significantly improved the preventive and therapeutic effects of MSCs against multiple diseases. We also describe the underlying mechanisms of the effects of RSV on the survival, self-renewal, and lineage commitment of MSCs in vitro and in vivo (Fig. 1). We showed that the use of the optimal dose of RSV may contribute greatly to MSC-based regenerative medicine.

## The administration and bioactivity of RSV

In general, people receive RSV via oral administration and intravenous administration, and the major route of RSV administration is oral intake. Due to its lipophilic features, RSV can be dissolved in alcoholic solutions, fruit juices, or vegetable juices or placed in capsules [18, 19].

Interestingly, RSV attracted the attention of many scientists because of a special phenomenon called the “French paradox” [15]. Despite the high-fat consumption of the French population, they have a low incidence of cardiovascular diseases since they frequently drink RSV-containing wine. A total of 50–100 mg/g of RSV exists in fresh grape skins, and approximately 0.2–5.8 mg/L of RSV exists in wine depending on the kind of wine [15]. After oral consumption, RSV is rapidly absorbed, and the plasma RSV concentration peaks at 30 min. Approximately 70–75% of the absorption occurs through transepithelial diffusion [20]. Since RSV is mainly metabolized in the intestine and liver through glucuronidation and sulfation, RSV bioavailability is highly reduced in vivo [21]. Although RSV has a high oral absorbance after digestion, its bioavailability is quite low due to its rapid metabolism [22, 23]. RSV is present as piceid in dietary products and can be metabolized by polyphenol oxidation, while glycosylation prevents the enzymatic oxidation of RSV and maintains its biological stability and bioavailability [24]. Thus, it is necessary to improve the bioavailability of RSV for MSC-based regenerative medicine.

Various RSV derivatives, such as prodrugs and synthetic analogs, have been developed to improve the bioavailability of RSV in vivo via the regulation of absorption, distribution, metabolism, and excretion and to ensure its biological and pharmacological activity without cytoxicity [25, 26]. In addition, other studies have proposed the use of different carriers to deliver increased levels of RSV into targeted tissues by improving the solubility, stability, permeability, and release of RSV in vivo [27]. Nanoformulations of RSV significantly enhance the absorption of RSV and protect it from the metabolism in animal models, and nanoparticle formulation was shown to ensure increased distribution in tissues such as the liver, heart, kidney, and ovary than more common methods of RSV administration [28]. However, most studies have used RSV but not RSV derivatives to improve the therapeutic effects of MSCs, and the optimization of delivery systems for RSV is more important for current MSC-based regenerative medicine. Thus, various delivery systems, including lipid nanocarriers, emulsions, micelles, polymeric nanoparticles, solid dispersions, and nanocrystals, have been developed for RSV encapsulation. These delivery systems protect RSV against degradation and ensure the sustained release of RSV. Lipid nanocarriers decreased the gastrointestinal damage of RSV and also improved the concentration and bioavailability of RSV in the brain, liver, and kidney of rats [29]. Subcellular-sized nanoemulsion systems also protect against chemical degradation and oxidation and then convert RSV into its *cis*-form [30]. Micellar solutions of bile acids that contained 3,7,12-triketocholic acids showed the smallest membranolytic potential, and they more effectively solubilized RSV and improved its resorption [31]. To further improve the oral bioavailability of RSV, polymeric nanoparticles that slowly degrade in vivo have been recognized as a more suitable oral delivery system than other rapidly degradable nanoparticles and nondegradable nanoparticles [32]. Recently, Chang et al. found that the easy manipulation and powdered form of solid dispersion drug delivery systems enhanced the solubility, dissolution, and absorption of RSV [33]. Moreover, nanoparticles could also be used to deliver RSV nanocrystals to further improve the solubility, dissolution rate, stability, compatibility, and oral bioavailability of RSV [34]. On the other hand, the intravenous administration of RSV completely bypassed gastrointestinal absorption and delivered RSV directly into serum to maximize the bioactivity of RSV. In comparison with oral administration, the intravenous administration of RSV and its metabolites led to a higher serum area under the curve in rats [35]. However, there are still no effective methods that can completely protect the bioactivity of RSV in vivo.

## In vitro effects of RSV on MSCs

To acquire a sufficient number of MSCs, MSCs are cultured ex vivo with animal serum and various growth factors; thus, we believe that repeated freezing/thawing, ex vivo culture conditions, and successive passaging are deleterious to MSCs and render them more susceptible to the microenvironment [36, 37]. The oxygen concentration of cultured MSCs in vitro (20–21% O_2_) is higher than that (i.e., 1–5% O_2_) encountered by MSCs tissues [38], and the difference in the surrounding oxygen concentration will consequently decrease the proliferation, differentiation, and anti-inflammatory effects of MSCs [39, 40]. RSV has been shown to protect MSCs from senescence and aging; moreover, RSV also participates in the regulation of the osteogenesis, adipogenesis, and neurogenesis of MSCs in vitro (Table 1).

**Table 1:** Various concentrations of RSV exert different effects on MSCs in vitro

RSV concentration	MSC source	Matrix	Mechanism	Effect	Reference
200 μM	Umbilical cord blood	N/A	Activate SIRT1	Attenuate IL-1β and NLRP3 expression induced by radiation	[41]
1 μM	Bone marrow	N/A	Activate SIRT1 but decrease β-catenin activity, ERK phosphorylation, and GSK-3β phosphorylation	Improve the self-renewal potential and multipotency of early passage MSCs	[42]
1 μM	Bone marrow	N/A	Decrease SIRT1 but increase β-catenin activity, ERK phosphorylation, and GSK-3β phosphorylation	Increase cellular senescence in late passage MSCs	[42]
0.1, 1, and 2.5 μM	Umbilical cord	N/A	Increase SIRT1 level while inhibiting the expression of p53 and p16	Promote cell viability and mitigate the senescence of MSCs	[43]
5 and 10 μM	Umbilical cord	N/A	Inhibit SIRT1 and PCNA and stimulate the expression of p53 and p16	Increase the levels of senescence and apoptosis in MSCs	[43]
60 μM	Menstrual blood	N/A	Reduce oxidative damage in quiescent MSCs and maintain physiological levels of reactive oxygen species in proliferating cells	Induce the reversible blockage of cell proliferation without genotoxic effects in quiescent MSCs and induce irreversible cell cycle arrest, DNA damage, and premature senescence in proliferating MSCs	[44]
10(-8)-10(-6) M	Bone marrow	N/A	Increase NO production and cGMP content	Increase cell growth and the osteogenic differentiation of MSCs	[45]
10^−6^ M	Bone marrow	N/A	Activate ERK1/2 and p38 MAPK	Increase cell growth and the osteogenic differentiation of MSCs	[46]
25 μM	Adipose tissue	Collagen-containing RSV scaffolds	Mimic in vivo microenvironment	Increase the level of mineralized matrix in the continuously treated group	[47]
50 μM	Adipose tissue	Collagen-containing RSV scaffolds	Enhance the epithelial and osteogenic differentiation of MSCs	Repair defects in calvarial bone	[48]
10 μM	Bone marrow	N/A	Upregulate the expression of mitofilin	Improve the osteogenic differentiation of senescent MSCs	[49]
10 nM	Periodontal ligament	N/A	Decrease p-NFκB p65 expression and rescue the p-AMPK level	Rescue the impairment of osteogenesis and regeneration in MSCs from periodontitis patients and normal MSCs treated with TNF-α	[50]
1 μM	Bone marrow	N/A	Upregulate hedgehog signaling	Reduce free radical production and protect against CSE-induced injury	[51]
25 μM	Bone marrow	N/A	Substitute for insulin in adipogenic medium and enhance the phosphorylation of cyclic AMP response element-binding protein (CREB)	Induce the robust adipogenesis of MSCs	[52]
1 μM	Bone marrow	N/A	N/A	Increase the expression of neuronal marker proteins and the number and length of neurites	[53]
2.5, 5, and 10 μM	Umbilical cord	N/A	Reduce the expression of nestin while upregulating the expression of βIII-tubulin and NSE in a dose-dependent manner and enhance the expression of neurogenin 1 and 2 as well as Mash1	Increase the neuronal differentiation of MSCs	[43]
10 μM	Cord blood	N/A	Increase the levels of protein kinase A, GSK-3β, and ERK1/2	Enhance the phosphorylation of CREB and increase the expression of neural markers	[54]
15 μM	Dental pulp	N/A	Increase the expression of the neuronal-specific marker genes nestin, musashi, and NF-M in MSCs	Promote the neuronal cell differentiation of MSCs	[55]
15.0 and 30.0 mg/L	Umbilical cord	N/A	N/A	Induce the differentiation of hUC-MSCs into neuron-like cells	[56]
1 μM	Bone marrow	N/A	Upregulate AMPK/SIRT1 signaling	Increase the levels of neuroprogenitor markers in MSCs isolated from ALS patients	[57]
10 μM	Cord blood	N/A	Activate the PI3K signaling pathway	Restore the impaired neuronal differentiation ability of MSCs induced by the neurotoxic organophosphate pesticide monocrotophos	[58]
10 μM	Cord blood	N/A	Activate the PI3K-mediated pathway	Repair monocrotophos-induced damage and protect against organophosphate pesticide-induced neurodegeneration	[58]

### The effects of RSV on MSC senescence and aging

Long-term in vitro cultures may lead to cell aging in MSCs accompanied by decreased self-renewal, increased cell senescence, upregulated cell apoptosis, and premature differentiation, consequently decreasing the therapeutic effects of MSCs in vivo. Pretreatment with RSV inhibited radiation-induced IL-1β expression by activating SIRT1 in a concentration-dependent manner, thus protecting cells against radiation-induced injury [41]. In contrast, there is a debate regarding the protective effects of RSV on aging and senescence in MSCs under other specific conditions. Although RSV effectively improved the self-renewal potential and multipotency of early passage MSCs via activating SIRT1 and decreasing β-catenin activity, ERK phosphorylation, and GSK-3β phosphorylation, it significantly increased the cellular senescence in late passage MSCs via the abrogation of the above pathways [42]. At concentrations of 0.1, 1, and 2.5 μM, RSV increased SIRT1 levels while inhibiting the expression of p53 and p16, thus promoting cell viability and mitigating the senescence of MSCs. RSV at concentrations of 5 and 10 μM increased the levels of senescence and apoptosis via inhibiting SIRT1 and PCNA and stimulating the expression of p53 and p16 [43]. A high dose of RSV (60 μM) is able to protect quiescent MSCs from oxidative damage and trigger the reversible blockage of cell proliferation without genotoxic effects, but this results in irreversible cell cycle arrest, DNA damage, and premature senescence in proliferating MSCs [44]. Although multiple studies have shown the protective effects of RSV, RSV will also exert cytotoxic effects on proliferating MSCs in vitro, which will lead to cell death.

### The effects of RSV on the osteogenic differentiation and adipogenic differentiation of MSCs

The use of RSV (10(-8)-10(-6) M) resulted in a dose-dependent increase in the growth and osteogenic differentiation of MSCs by increasing NO production and cGMP content [45] and activating ER-mediated extracellular signal-regulated kinase 1/2 (ERK1/2) [46]. MSCs showed good adherence to collagen-containing RSV (collagen/RSV) scaffolds, and pretreatment with 25 μM RSV for a short time increased the amount of mineralized matrix in the continuously treated group [47]. In addition, MSCs cultured on collagen/RSV scaffolds demonstrated a higher proliferation rate than those cultured on collagen scaffolds. Defects in the calvarial bone could be repaired more effectively by MSCs on collagen/RSV scaffolds than by MSCs on collagen scaffolds [48].

In addition to normal MSCs, RSV can upregulate the osteogenic differentiation of MSCs in some pathological conditions. RSV significantly upregulated the expression of mitofilin, which is the core component of the mitochondrial contact site and cristae organizing system (MICOS), and then enhanced the osteogenic differentiation of senescent MSCs in vitro [49]. RSV rescued the impairment of osteogenesis and regeneration in MSCs from periodontitis patients and in normal MSCs treated with TNF-α [50]. Cigarette smoke extract (CSE) impaired the primary cilia distribution and osteogenic differentiation of MSCs via downregulating hedgehog signaling, while 1 μM RSV significantly reduced free radical production and protected against CSE-induced injury [51].

In addition, 1 μM RSV enhanced the adipogenic or osteogenic differentiation, and 25 μM RSV resulted in the robust adipogenesis of MSCs upon substitution for insulin in adipogenic medium and enhanced the phosphorylation of cyclic AMP response element-binding protein (CREB) [52].

Therefore, RSV may participate in the regulation of osteogenesis and adipogenesis of MSCs in vitro, as this indicates that the number and quality of studies about the effects of RSV on the adipogenesis of MSCs should be improved. RSV may act as an antioxidant or nutritional compound during the differentiation process, and its specific effects should be further determined according to its concentration.

### The effects of RSV on the neural differentiation of MSCs

Recent studies have demonstrated that RSV concentrations within a wide range are safe and able to promote MSC neurogenesis in vitro. Although untreated MSCs and MSCs pretreated with 1 μM RSV exhibited similar morphology to the neurons after culture in neuronal induction media, the RSV-pretreated MSCs showed a significant increase in the expression of neuronal marker proteins and the number and length of neurites when compared with untreated MSCs [53]. At concentrations of 2.5, 5, and 10 μM, RSV increased the neuronal differentiation of human MSCs in a dose-dependent manner via reducing the expression of nestin and upregulating the expression of βIII-tubulin, NSE, neurogenin, and mash1 [43]. A concentration of 10 μM RSV alone or in combination with nerve growth factor enhanced the phosphorylation of CREB and increased the expression of neural markers via the activation of protein kinase A, GSK-3β, and ERK1/2 [54]. RSV (15 μM) increased the expression of neuron-specific marker genes such as nestin, musashi, and NF-M in MSCs, thus promoting the neuronal cell differentiation of MSCs [55]. Intriguingly, Guo et al. demonstrated that a high dose of RSV (30 mg/L) exerted no negative effect on GFAP and glial markers in MSCs and also significantly induced the differentiation of MSCs into neuron-like cells [56].

MSCs from amyotrophic lateral sclerosis (ALS) patients have functional limitations in terms of the release of neurotrophic factors and exhibit a senescent phenotype. Pretreatment with 1 μM RSV increased the levels of neuroprogenitor markers in MSCs isolated from ALS patients via the upregulation of AMPK/SIRT1 signaling. The differentiated ALS-derived MSCs exhibited a cell body and dendritic shape similar to neurons [57]. RSV at 10 μM successfully restored the alterations of MSCs and rescued the impaired neuronal differentiation ability of MSCs induced by the neurotoxic organophosphate pesticide monocrotophos [58]. Moreover, 10 μM RSV repaired monocrotophos-induced damage via the PI3K-mediated pathway and promoted the neuronal differentiation of MSCs, thus indicating potential protection by RSV against organophosphate pesticide-induced neurodegeneration [58].

## The effects of RSV on MSCs in vivo

A harsh microenvironment in vivo induces MSCs to undergo senescence or apoptosis, thus impairing the balance between ROS and antioxidant mechanisms and decreasing the therapeutic effects of MSCs by decreasing their homing, differentiation, and paracrine effects [59–61]. The optimization of MSC culture conditions can serve as a key strategy to improve MSC functioning in vitro and in vivo, and RSV may serve as an effective agent for protecting MSCs from oxidative stress (Table 2).

**Table 2:** Various concentrations of RSV exert different effects on MSCs in vivo

RSV dosage	MSC source	MSC dose	Injury/disease	Model	Mechanism	Effect	Reference
10 mg/kg	Bone marrow	1 × 10^6^	CBDL	Rat	Upregulate the level of SIRT1 and downregulate the level of p53, upregulate the homing of MSCs to the liver, and decrease the homing of MSCs to the lung and spleen	Eliminate liver cirrhosis	[62]
100 mg/kg	Bone marrow	1 × 10^6^	Partial hepatectomy	Rat	Increase the homing of MSCs to the liver	Enhance liver regeneration	[63]
200 mg/kg	Umbilical cord	1 × 10^6^	Transgenic Alzheimer’s disease mouse model	Mouse	Increase the engraftment of MSCs	Improve learning and memory, enhance neurogenesis, and alleviate neural apoptosis in the hippocampus in an Alzheimer’s disease mouse model	[64]
200 mg/kg	Umbilical cord	1 × 10^6^	Alzheimer’s disease	Mouse	Increase the expression of hippocampal SIRT1, PCNA, p53, ac-p53, p21, and p16	Increase the neurogenesis of MSCs	[64]
100 mg/kg	Adipose tissue	2 × 10^6^/kg	Doxorubicin-induced injury	Rat	Enhance the cardiogenic differentiation and paracrine effects of MSCs	Prevent doxorubicin-induced cardiomyopathy	[65]
20 μM (pretreatment)	Umbilical cord	1 × 10^6^	Cisplatin-induced injury	Rat	Upregulate the secretion of PDGF-DD and increase the phosphorylation of ERK	Downregulate the apoptosis of renal tubular cells, upregulate the angiogenesis of endothelial cells, and decrease kidney injury	[66]
30 mg/kg	Bone marrow	1.5 × 10^6^	*Mycobacterium tuberculosis* H37Ra and pertussis toxin injury	Mouse	Suppress the release of proinflammatory cytokines (IFN-γ and TNF-α) and increase the release of anti-inflammatory cytokines (IL-4 and IL-10)	Reduce the clinical scores of patients with autoimmune encephalomyelitis	[67]

### The effects of RSV on MSC engraftment

Although MSCs may form strategic niches in perivascular spaces in almost every region of the body, they migrate to sites of injury for inflammation suppression and wound healing after the detection of local and distant tissue damage [68, 69]. However, the injection of MSCs through the blood vessels leads to the engraftment of MSCs into the lungs and the capillary beds of other tissues or organs, consequently decreasing the number of MSCs migrating to the target areas [70]. RSV pretreatment in a common bile duct ligation (CBDL) animal model improved the therapeutic effects of MSCs via the upregulation of SIRT1 and the downregulation of p53, accompanied by the upregulation of the homing of MSCs to the liver and a decrease in homing of MSCs to the lung and spleen [62]. In rats with partial hepatectomy, RSV significantly increased the homing of MSCs to the liver and enhanced liver regeneration [63]. RSV also increased the engraftment of MSCs for the repair of hippocampal injuries in an Alzheimer’s disease mouse model, thus improving learning and memory, enhancing neurogenesis, and alleviating neural apoptosis [64].

### The effect of RSV on differentiation in vivo

Various dosages of RSV (1, 5, and 10 mg/kg) significantly decreased myocardial lesions by increasing myocardial AKT expression and decreasing caspase-3 activity during carbon monoxide-induced cardiotoxicity in rats in a dose-dependent manner [71]. RSV-pretreated MSCs showed increased proliferation and demonstrated enhanced cardiac remodeling capacity in diabetic cardiomyopathy via the attenuation of secreted frizzled-related protein-mediated fibrosis and the Wnt/β-catenin pathway [72]. The combination of RSV exposure and MSC transplantation successfully prevented doxorubicin-induced cardiomyopathy in rats via enhancing the cardiogenic differentiation and the paracrine effects of MSCs [65]. Wang et al. demonstrated that RSV increased the neurogenesis of MSCs by increasing the expression of hippocampal SIRT1, PCNA, p53, ac-p53, p21, and p16 in the hippocampus in an Alzheimer’s disease mouse model [64]. A concentration of 20 μM RSV upregulated the secretion of platelet-derived growth factor-DD (PDGF-DD) and increased the phosphorylation of ERK, thus downregulating the apoptosis of renal tubular cells. In addition, the release of the cytokine PDGF-DD upregulated the angiogenesis of endothelial cells; thus, the administration of RSV served as a novel strategy for enhancing the therapeutic efficacy of MSCs in cisplatin-induced kidney injury [66].

### The effect of RSV on paracrine mechanisms

Interestingly, apart from the engraftment and differentiation of MSCs in vivo, initial preclinical animal models of inflammatory conditions suggested that MSCs protected against tissue injury via various paracrine mechanisms. MSCs exert paracrine effects via the release of multiple immunomodulatory factors via microvesicles, microRNAs, exosomes, and mitochondrial transfer [73]. RSV significantly decreased the expression of TNFα-induced inflammatory cytokines, including IL-6 and IL-8, in MSCs after the activation of autophagy and the inhibition of the JNK signaling cascade [74]. In addition, the combination of RSV exposure and MSC transplantation significantly reduced the clinical scores of patients with autoimmune encephalomyelitis via suppressing the release of proinflammatory cytokines (IFN-γ and TNF-α) and increasing the release of anti-inflammatory cytokines (IL-4 and IL-10) [67].

## Conclusion

RSV participates in regulating the survival, self-renewal, and multipotency of MSCs in vitro prior to MSC therapy. However, the concentration, time of administration, and duration of RSV pretreatment will influence the effects of RSV on MSCs in vitro and in vivo. We believe that further studies should focus on the therapeutic effects of RSV and MSC transplantation on diseases of the organs in addition to the liver, brain, heart, and kidney. On the other hand, although there are multiple RSV derivatives, including methoxylated, hydroxylated, and halogenated forms, that protect against multiple diseases, the application of RSV derivatives to MSC-based regeneration has been subject to little study; thus, it is necessary to investigate the protective effects of RSV derivatives in future studies. Furthermore, although RSV is safe and has been tolerated at dosages of up to 5 g/day without major side effects in long-term clinical trials [22, 75], the intake of 2.5 g or more per day of RSV may produce side effects such as nausea, vomiting, diarrhea, and liver dysfunction in patients with nonalcoholic fatty liver disease [76]. Therefore, the side effects of RSV may vary in sick patients; thus, we conclude that RSV is relatively safe for clinical application. It is worth noting that RSV will lose its bioactivity in vivo after oral or transvenous administration; thus, new biomaterials that can greatly improve the bioactivity or release of RSV will certainly contribute to MSC-based regenerative medicine.

## Data Availability

All data are included in this published article.

## Abbreviations

MSCs: Mesenchymal stromal cells
RSV: Resveratrol
ROS: Reactive oxygen species
RNS: Reactive nitrogen species
ERK1/2: Extracellular signal-regulated kinase 1/2
MICOS: Mitochondrial contact site and cristae organizing system
CSE: Cigarette smoke extract
CREB: Element-binding protein
ALS: Amyotrophic lateral sclerosis
CBDL: Common bile duct ligation
PDGF-DD: Platelet-derived growth factor-DD
